# Diagnostic value of repeated Dix-Hallpike and roll maneuvers in benign paroxysmal positional vertigo^☆^

**DOI:** 10.1016/j.bjorl.2016.03.007

**Published:** 2016-04-22

**Authors:** Cenk Evren, Nevzat Demirbilek, Mustafa Suphi Elbistanlı, Füruzan Köktürk, Mustafa Çelik

**Affiliations:** a Medilife Beylikduzu Hospital, Department of Otolaryngology-Head and Neck Surgery, Istanbul, Turkey; b Bakirkoy Dr. Sadi Konuk Training and Research Hospital, Department of Otolaryngology-Head and Neck Surgery, Istanbul, Turkey; c Bülent Ecevit University, Faculty of Medicine, Department of Biostatistics, Zonguldak, Turkey

**Keywords:** Dix-Hallpike maneuver, Repeat, Vertigo, Manobra de Dix-Hallpike, Repetição, Vertigem

## Abstract

**Introduction:**

Benign Paroxysmal Positional Vertigo (BPPV) is the most common peripheral vestibular disorder. The Dix-Hallpike and Roll maneuvers are used to diagnose BPPV.

**Objective:**

This study aims to investigate the diagnostic value of repeated Dix-Hallpike and Roll maneuvers in BPPV.

**Methods:**

We performed Dix-Hallpike and roll maneuvers in patients who admitted with peripheral vertigo anamnesis and met our criteria. The present study consists of 207 patients ranging in age from 16 to 70 (52.67 ± 10.67). We conducted the same maneuvers sequentially one more time in patients with negative results. We detected patients who had negative results in first maneuver and later developed symptom and nystagmus. We evaluated post-treatment success and patient satisfaction by performing Dizziness Handicap Inventory (DHI) at first admittance and two weeks after treatment in all patients with BPPV.

**Results:**

Of a total of 207 patients, we diagnosed 139 in first maneuver. We diagnosed 28 more patients in sequentially performed maneuvers. The remaining 40 patients were referred to imaging. There was a significant difference between pre- and post-treatment DHI scores in patients with BPPV (*p* < 0.001).

**Conclusion:**

Performing the diagnostic maneuvers only one more time in vertigo patients in the first clinical evaluation increases the diagnosis success in BPPV. Canalith repositioning maneuvers are effective and satisfactory treatment methods in BPPV.

## Resumo

**Introdução:**

Vertigem posicional paroxística benigna (VPPB) é a disfunção vestibular periférica mais comum. As manobras de Dix-Hallpike e *roll-test* são usadas para diagnosticar a VPPB.

**Objetivo:**

Este estudo teve como objetivo investigar o valor diagnóstico da repetição das manobras de Dix-Hallpike e *roll-test* na VPPB.

**Método:**

Manobras de Dix-Hallpike e *Roll-test* foram realizadas nos pacientes que foram internados com história de vertigem periférica e eram adequados aos nossos critérios. O presente estudo contou com 207 pacientes na faixa etária de 16-70 anos (52,67 ± 10,67). Fizemos uma vez mais as mesmas manobras sequencialmente nos pacientes com resultados negativos. Detectamos os pacientes que tiveram resultados negativos na primeira manobra e que posteriormente desenvolveram sintomas e nistagmo. Avaliamos o sucesso pós-tratamento e a satisfação do paciente mediante o Inventário da Deficiência Física na Vertigem (*Dizziness Handicap Inventory* - DHI) na primeira admissão e duas semanas após o tratamento em todos os pacientes com VPPB.

**Resultados:**

De 207 pacientes, 139 foram diagnosticados na primeira manobra. Diagnosticamos mais 28 pacientes nas manobras feitas consecutivamente. Os 40 pacientes restantes foram encaminhados para exames de imagem. Houve diferença significativa entre os escores do DHI pré- e pós-tratamento nos pacientes com VPPB (*p* < 0,001).

**Conclusão:**

A realização das manobras diagnósticas apenas mais uma vez nos pacientes com vertigem na primeira avaliação clínica aumentou o sucesso do diagnóstico em VPPB. As manobras de reposicionamento canalicular são métodos eficazes e satisfatórios de tratamento na VPPB.

## Introduction

Vertigo is separated into two as vertigo of central or peripheral origins. More than 90% of vertigo consists of Benign Paroxysmal Positional Vertigo (BPPV), acute peripheral vestibulopathy, and Meniere disease. Central vertigo is accompanied by neurologic symptoms such as diplopia, dysarthria, incoordination, drowsiness, and weakness. It is milder but lasts longer. Nystagmus resulting from positional maneuvers in central lesions has no latency or fatigue as in BPPV, appears immediately after positional maneuvers, and at the same amplitude and frequency in repeated maneuvers.^1^, ^2^

Benign paroxysmal positional vertigo is defined as dizziness which may last for a few seconds or up to one minute due to sudden movements of the head and accompanying nystagmus. It is the most commonly observed peripheral vestibular disorder in the practice of ear, nose, and throat.^3^ Diagnosis of BPPV, which decreases quality of life considerably and is a common disorder, can be established by anamnesis and detection of positional nystagmus.

Certain theories exist regarding the development of BPPV. Schuknecht supports the cupulolithiasis theory, which is based on the attachment of otolithic debris to the cupula in crista ampullaris.^4^ Hall et al. propose the theory of canalithiasis, which is based on free-floating debris in the canal.^5^ Both these theories support the presence of foreign particles in semisirculer canal as a cause of vertigo.^5^

Detection of the involved canal in BPPV is important in terms of the treatment to be performed. To establish a diagnosis of posterior canal BPPV, characteristic nystagmus should be confirmed by the Dix-Hallpike (DH) maneuver and this is one of the diagnostic criteria.^6^ Whereas supine head roll maneuver (Pagnini-McClure maneuver) is used to demonstrate horizontal canal BPPV. Generally, only one maneuver can be performed in patients in polyclinic conditions.^3^, ^6^, ^7^, ^8^, ^9^

Patients with positive results after diagnostic maneuvers are administered appropriate treatment maneuvers while patients with negative maneuver results are referred to other branches considering central or internal reasons even if their anamneses are peripheral.^3^, ^6^, ^7^, ^8^, ^9^ More invasive and costly additional examinations are requested to establish a diagnosis including Magnetic Resonance Imaging (MRI), Computerized Tomography (CT), Doppler, Electronystagmography (ENG), Videonystagmography (VNG), bithermal caloric maneuver, etc.^8^, ^9^

Many studies have reported incorrect negative results with DH maneuver.^10^, ^11^, ^12^, ^13^, ^14^ Viirre et al. have indicated that, in 10%–20% of patients with no symptom or examination finding after DH and roll maneuvers, diagnosis was established with sequential repetition of DH maneuver.^12^ They suggested that this condition was probably due to debris that was dispersed throughout the posterior canal forming a clot that is more effective in displacing the cupula during the brief period of lying supine. For whatever reason, the simple procedure of repeating the DH maneuver after the horizontal maneuver has increased positivity.^12^

In this study, we aimed to investigate the diagnostic value of repeated Dix-Hallpike and Roll maneuvers in BPPV.

## Methods

We prospectively evaluated 207 patients (52.67 ± 10.67) aged between 16 and 70 who admitted to our Hospital Ear Nose Throat Clinic between December 2013 and March 2015 describing positional vertigo. The study protocol was approved by the ethical board of the hospital (no. 104).

We questioned the duration and type of vertigo, any accompanying hearing loss, tinnitus, aural fullness, neurological deficit concomitant of the attacks (facial paralysis, mental haziness, power loss, syncope, etc.), systemic disease, continuous drug use, and history of trauma. All patients were performed pure tone audiometry and stapedius reflex test so as not to omit any additional middle-inner ear pathology.

Patients with gaze-evoked nystagmus (30° horizontally and vertically), positive result of the DH maneuver in both right and left head-hanging positions, evidence of ongoing central nervous system disease (e.g. transient ischemic attack), otitis media, otosclerosis, vestibular complaints other than positional vertigo, and patients who were unable to tolerate DH maneuver were excluded.

All patients underwent DH and roll maneuver following ear–nose–throat and neurological evaluations. Frenzel glasses were used in all patients.

The first DH maneuver was performed with a clinical examination chair extended into the horizontal position. Patients were seated upright, their heads turned 45° to either right or left, and then positioned flat with extension of the head on the neck. The diagnosis of BPPV required a positive DH maneuver with the following criteria: 1) brief latency between the onset of nystagmus and vertigo and head positioning, and 2) observation of a paroxysmal upbeating and torsional (fast component of the superior pole of the eye beating toward the undermost ear) nystagmus associated with a perception of vertigo.^15^

Patients with negative DH maneuver results were performed roll maneuver subsequently. Roll maneuver was performed in supine position, with the head fixed at 30° of flexion and rotated to right or left. Patients with geotropic or ageotropic horizontal nystagmus were diagnosed with horizontal canal BPPV.^16^

Patients with positive results in first DH and roll maneuvers were named as Group 1. Patients describing positional vertigo and with negative DH and roll maneuver results were immediately (after 30 seconds) performed second DH and roll maneuvers. Results were recorded. Patients with positive results in second DH and roll maneuvers were named as Group 2.

Patients with positive maneuver results were performed Epley maneuver for the posterior canal and barbeque maneuver for the horizontal canal. None of the patients were administered bone vibrator or drug treatment.

Patients with negative maneuver results were referred to MRI examination to distinguish any organic lesions in their central nervous systems. All patients were required to complete the Turkish version of the Dizziness Handicap Inventory (DHI) before maneuver. Two to three days after the Epley and barbeque maneuver procedures, DH and roll maneuvers were repeated for control purposes. Maneuvers were repeated in patients with ongoing positivity in two to three day intervals and for a maximum of three times for one side. Recovery was considered with improved symptoms and negativity in control DH and roll maneuvers. All patients were required to recomplete the DHI after two weeks from first arrival. Patients with negative maneuver results were not required to complete the DHI.

Statistical analyses were performed with SPSS 19.0 software (SPSS Inc., Chicago, IL, USA). Descriptive statistics were expressed as frequency and percentage. The Chi-square test was used to determine differences between groups. Related measures were evaluated with the McNemar's test. Paired samples *t*-test was used to compare pre- and post-treatment DHI results. A *p*-value of less than 0.05 was considered statistically significant for all tests.

## Result

Patients’ female to male ratio (Table 1) and demographic data (Table 2) were recorded. A total of 207 patients (98 males, 109 females) admitted with complaint of vertigo.

**Table 1:** Female-male ratios.

Valid	Frequency	Percentage	Valid percentage	Cumulative percentage
Male	98	47.3	47.3	47.3
Female	109	52.7	52.7	52.7

Total	207	100.0	100.0

**Table 2:** Clinical and demographic characteristics of patients with BPPV.

Affected side (right/left)	1/1.5
Affected canal	Posterior canal 97.7%
	Horizontal canal 2.3%
Duration of vertigo (days)	6.3 ± 9.8
Etiology	Trauma 17.3%, idiopathic 82.7%
First episode of vertigo	72%

Of the 207 patients, 135 (65%; 58 males, 77 females) had positive results in the first DH maneuver. These 135 patients were directed to Epley maneuver. The remaining 72 patients were performed roll maneuver. Four patients (2%; 3 males, 1 female) with positive results were directed to barbeque maneuver. In other words, of the 207 patients, we diagnosed 139 in the first DH and roll maneuvers and treated them (Group 1).

We repeated the DH maneuver immediately after the first DH and roll maneuvers in 68 patients with negative results. Of these patients, 28 (13 males, 15 females) had positive results. No patient had positive result after repeating the roll maneuver (Group 2). Forty patients with negative results in all maneuvers were directed to MRI. No organic pathology was detected in any of these patients.

As a result, we diagnosed 135 patients (65%) in the first DH maneuver and four patients (2%) in the first roll maneuver. Of the remaining 68 patients, we diagnosed 28 in the second DH maneuver. This rate was 13% of the grand total and 41% of the remaining 68 patients.

Of the 207 patients, we detected BPPV in 139 in first maneuver and in 167 in second maneuvers. A comparison of the groups revealed a significant difference (*p* < 0.001) (Table 3).

**Table 3:** Comparison of groups.

		Group 1		Total
		−	+	
*Group 2*				
−	Count	40	139	179
	% within group 2	21.3	78.7	100.0
	% within group 1	55.5	100.0	86.5
+	Count	28	0	28
	% within group 2	100.0	0.0	100.0
	% within group 1	38.9	0.0	13.5

*Total*				
	Count	68	139	207
	% within group 2	32.8	67.2	100.0
	% within group 1	100.0	100.0	100.0

An analysis of the relationship between age groups showed no significant difference between patients aged below and above 50 in terms of the results of Group 1 (*p* = 0.748) and Group 2 (*p* = 0.378).

A review of the relationship between sexes demonstrated no significant difference between males and females in terms of the results of Group 1 (*p* = 0.084) and Group 2 (*p* = 1.000).

A comparison of total DHI scores before maneuvers and after recovery revealed a statistically significant improvement in patients (*p* < 0.001) (Table 4).

**Table 4:** Difference between groups regarding general subscale Dizziness Handicap Inventory score.

	Pre-treatment total DHI average	Post-treatment total DHI average	
Group 1	64.85	12.35	*p* < 0.001
Group 2	60.20	10.85	*p* < 0.001

DHI, Dizziness Handicap Inventory.

No relapse was detected during the three-month follow-up of patients who were performed Epley and barbeque maneuvers. No complication developed.

## Discussion

Benign paroxysmal positional vertigo is the most common type of dizziness in general practice. It is responsible for up to 25% of all instances of vertigo. BPPV is generally observed in fifth and sixth decades. It is the most common cause of vertigo after a head injury.^17^, ^18^

Patients with BPPV most commonly report discrete, episodic periods of vertigo lasting 1 minute or less and often modifications or limitations of their general movements to avoid provoking the vertiginous episodes.^19^ Other symptoms of BPPV include disequilibrium, increased risk of falling and fear of falling, light headedness, nausea, decreased activity levels, anxiety, impaired vision, headache, and vomiting.^11^, ^12^, ^13^, ^14^, ^15^, ^16^, ^17^, ^18^

The episodes are often provoked by everyday activities and commonly occur when rolling over in bed or tilting the head to look upward (e.g. to place an object on a shelf higher than the head) or bending forward (e.g. to tie shoes).^13^, ^18^

Detection of the involved canal in BPPV is important in terms of the treatment to be performed. When the pathology is identified, success rates may increase in first maneuver with the appropriate treatment.^20^ While DH is maneuver is used to detect posterior canal involvement, roll maneuver is used to detect horizontal canal involvement.^8^, ^9^, ^11^, ^12^, ^13^

Since posterior canal involvement is frequently observed or involvement of posterior or superior canals is considered based on history and existence of rotatory nystagmus in a case with suspected BPPV, diagnosis should be first attempted to be made with maneuver. The DH maneuver is the gold standard for diagnosing BPPV.^11^, ^13^, ^18^

Posterior canal BPPV has been said to account for 60%–90% of all BPPV cases, and horizontal canal BPPV (also called Lateral canal BPPV) for 5%–30% of the cases.^21^, ^22^ Horizontal and anterior canal variants are less prevalent because they are not in a gravity-dependent position. In a study by Cakir et al., posterior canal BPPV was the confirmed diagnosis in 144 (85.2%), horizontal canal BPPV in 20 (11.8%) and anterior canal BPPV in 2 (1.2%) patients.^22^ In our study, we detected posterior canal involvement in 97.7% and horizontal canal involvement in 2.3% of all patients. No anterior canal involvement was identified.

Therefore, a negative DH maneuver does not necessarily rule out the diagnosis of posterior canal BPPV. Because of the lower negative predictive values of the DH maneuver, it has been suggested that this maneuver may need to be repeated at a separate visit to confirm the diagnosis and avoid a false-negative result. In a study, Lopez-Escamez et al. indicated a sensitivity of 82% and specificity of 71% in DH maneuver in posterior canal BPPV.^23^ Hanley and O’dowd stated a positive predictive value of 83% and a negative predictive value of 52%.^17^ For this reason, a negative result in DH maneuver does not eliminate a diagnosis of posterior canal BPPV.^10^, ^12^, ^24^

Factors affecting the diagnostic value of DH maneuver include the speed of movements during maneuver, the time of day, and the angle of the occipital plane during the maneuver.^24^ Burston et al. found no evidence that time of day affects the result of the DH maneuver when maneuvering for BPPV in a relatively unselected clinical population with a history of likely BPPV.^25^

In our study, we performed the maneuvers sequentially, resulting in positive results in 28 of 68 patients who had negative results in first maneuvers. In other words, we misevaluated 28 patients in first maneuvers. Similarly with Viirre et al., we believe that this condition was probably due to debris that was dispersed throughout the posterior canal forming a clot that is more effective in displacing the cupula during the brief period of lying supine.^12^

Brandt et al. indicated that inappropriately treated BPPV may continue for months.^26^ Therefore, it is important to make a correct diagnosis. In cases with suspected BPPV, canalith repositioning maneuver is the frequently attempted treatment. Successful results were achieved with one administration of Epley's canalith repositioning maneuver designed to repose the endolymphatic debris from the posterior semicircular canal into the vestibule or Semont's releasing maneuver.^24^, ^27^ Epley, Wolf, and Ruckenstein demonstrated success rates of 97%, 93.4%, and 70% with one maneuver, respectively.^4^, ^28^ CRP or modified Epley maneuver are usually ineffective for horizontal canal BPPV.^8^, ^22^, ^29^, ^30^ Variations of the roll maneuver (Lempert maneuver or barbecue roll maneuver) are the most widely published treatments for horizontal canal BPPV.^8^, ^22^, ^30^, ^31^

Dizziness Handicap Inventory is a 25 article scale evaluating functional, emotional, and physical quality of life in patients with vertigo and balance disorder.^32^, ^33^ DHI was reported to be a scale which may assist clinicians in the diagnosis and follow-up of patients with BPPV.^10^ Improved quality of life was observed in patients with BPPV after treatment with canalith repositioning maneuvers.^23^ Andre et al. showed that the Brazilian version of DHI administered before and after treatment in BPPV is beneficial in assessing treatment efficiency.^7^ In that study, the researchers obtained improved functional, emotional and physical parameters as well as quality of life with DHI in patients with BPPV. Similarly, in our study, an analysis of DHI results revealed significant improvement after maneuvers in both patient groups. We achieved lower DHI scores by performing the DH maneuver a second time in a vertigo population who had negative results in first DH maneuver.

The most common “complication” of BPPV repositioning treatment is canal conversion. Considering the population age in which it is usually performed, there is a surprising sparsity of literature on cervical spine and neurological complications.^29^, ^34^ In our study, patients were successfully treated without any complication.

Repeated DH maneuver in misdiagnosed patients may reduce the cost of the treatment by preventing unnecessary diagnostic tests. Patients may be diagnosed faster while costly and inconvenient additional examinations may not be required. Further studies are needed whether to see if repeated DH maneuver will reduce the cost of the treatment.

The fact that we did not use objective methods such as ENG or VNG may be a limitation of this study.

## Conclusion

Performing the diagnostic maneuvers only one more time in vertigo patients in the first clinical evaluation increases the diagnosis success in BPPV. Canalith repositioning maneuvers are effective and satisfactory treatment methods in BPPV.

## Conflicts of interest

The authors declare no conflicts of interest.
